# Factors influencing the acceptance of medical AI chat assistants among healthcare professionals and patients: a survey-based study in China

**DOI:** 10.3389/fpubh.2025.1637270

**Published:** 2025-09-04

**Authors:** Lanshan Zhang, Jingyi Yang, Gege Fang

**Affiliations:** ^1^School of Digital Media and Design Arts, Beijing University of Posts and Telecommunications, Beijing, China; ^2^New Media and Creative Center at the Beijing Key Laboratory of Internet Systems and Network Culture, Beijing, China

**Keywords:** medical, AI chat assistant, UTAUT model, artificial intelligence, digital competence, technological acceptance, China

## Abstract

**Introduction:**

This study examines the drivers behind healthcare professionals’ and patients’ acceptance of medical AI chat assistants in China.

**Methods:**

A nationwide online survey was conducted from March 10 to April 28, 2024, using quota sampling to collect 500 valid responses, and data were analyzed via structural equation modeling.

**Results:**

Performance expectancy, perceived cost, digital access, and digital competence all positively predicted intention to use, whereas patient age and consultation frequency—rather than socioeconomic status—directly influenced actual usage behavior.

**Discussion:**

Findings validate an extended UTAUT model in digital-health contexts, highlight cultural moderators of technology acceptance, and inform age-friendly AI design and equitable health-informatization policies in aging societies.

## 1 Introduction

As a populous nation with an outsized demand for healthcare, China recorded 9.54 billion outpatient visits in 2023—an average of seven encounters per capita (1). Although Artificial Intelligence-Generated Content technologies have been integrated into clinical workflows, longstanding asymmetries in resource allocation and persistent supply–demand imbalances remain significant (2). Despite incremental advances under the Healthy China 2030 agenda, the structural tension between service supply and population need endures.

The rapid maturation of artificial intelligence has catalyzed the emergence of “AI+Healthcare,” deepening algorithmic penetration across the care continuum and offering novel pathways to redress shortages and maldistribution of medical resources. Industry forecasts project that China’s AI application market will reach US$127 billion by 2025, with the medical AI segment accounting for one-fifth of the total value (3). AI systems are increasingly positioned as “second brains” for clinicians, assuming partial responsibility for resource provision and bearing the societal expectation of alleviating systemic strain.

The November 2022 release of ChatGPT accelerated the development of Chinese medical large language models and facilitated the rollout of AI-empowered conversational agents. In May 2023, MedLink unveiled MedGPT, its proprietary medical chatbot, which achieved a 96 percent concordance rate with face-to-face initial diagnoses in a controlled experiment (4). In October 2023, Huatuo GPT successfully passed the National Medical Licensing Examination and nearly all other medical credentialing tests. By November, the system had already served several hundred thousand users (5). These milestones underscore the substantial value and latent potential of AI chatbots within China’s health ecosystem.

However, technological breakthroughs have not been accompanied by commensurate adoption. In 2023, the urban AI adoption rate was only 6.3 percent, with fewer than 1 million AI-assisted consultations per day—an overall penetration rate below 4 percent. To meet the target set in the 14th Five-Year Plan for National Health Informatization—namely, achieving a 50 percent utilization rate of intelligent diagnostic aids in tertiary hospitals by 2025—a compound annual growth rate exceeding 35 percent is required over the next 24 months (6). This supply–demand asymmetry foregrounds the urgency of identifying the drivers of AI adoption.

Echoing Chen et al.’s systematic review, extant scholarship on AI in healthcare predominantly privileges technical dimensions or single-stakeholder perspectives—either patients or clinicians—thereby neglecting systematic analyses of acceptance gaps across the dyadic doctor–patient relationship (7). Moreover, most studies rely on the original Technology Acceptance Model (TAM) or the Unified Theory of Acceptance and Use of Technology (UTAUT) without incorporating context-specific variables pertinent to China’s medical environment (8). By extending UTAUT to incorporate Digital Access, Digital Competence, and patients’ Socio-Economic Status, this research offers a theoretically grounded and empirically nuanced account of the factors shaping the acceptance and use of AI-enabled chatbots among both clinicians and patients, ultimately informing policy and practice aimed at enhancing service delivery and doctor-patient relations.

## 2 Literature review

### 2.1 Application and effects of medical AI chat assistants

#### 2.1.1 Application status and ethical risks

Medical AI chat assistants represent a new form of healthcare technology developed within the context of large language models. These assistants enable patients to provide information such as their age, symptoms, and medical history through conversation, in exchange for receiving a diagnosis and treatment plan. Healthcare professionals can use these AI-generated assessments to validate or assist their own diagnostic and treatment decisions.

In terms of application, Nov et al. conducted a survey to explore respondents’ ability to recognize and trust medical advice provided by ChatGPT compared to human doctors. The findings indicated that ChatGPT’s accuracy in addressing patients’ medical questions ranged from 49 to 85%, with a minimal distinction rate between human and machine feedback (65.5% for humans vs. 65.1% for machines) (9). Moreover, the trust in ChatGPT’s medical responses was moderately positive. Additionally, Ayesha et al. investigated the potential of medical artificial intelligence in assisting healthcare professionals with diagnosing and treating cardiovascular diseases through an experimental approach. They posed 10 hypothetical clinical cardiovascular disease questions to ChatGPT and had the medical feedback evaluated by human medical experts. The results showed that eight of ChatGPT’s responses were accurate diagnoses, while the other two, although not entirely incorrect, had some discrepancies from the actual diagnostic procedures (10).

In terms of ethical risks, Liu highlighted significant concerns regarding the “technological black box” and the autonomous learning capabilities of medical AI. These risks include challenges in regulating the protection of medical data and difficulties in assigning accountability when medical harm occurs (11). On the other hand, Starke et al. developed a trust model based on research into human-machine interaction. They argued that despite the risks, medical AI can be considered trustworthy by focusing on its reliability, capability, and intent. They called for the cultivation of reasonable trust in medical AI (12).

#### 2.1.2 Impact on doctor-patient relationships and treatment outcomes

The impact of medical AI chat assistants on doctor-patient relationships has elicited mixed views within the academic community. Some scholars argue that medical AI chat assistants have a positive influence on doctor-patient relationships. Margam suggests that ChatGPT provides medical professionals with extensive medical knowledge, which helps them make more informed decisions and reduces administrative burdens, enabling them to focus on delivering compassionate and effective patient care (13). Similarly, Lu et al., based on evidence-based medicine principles, argue that AI can offer precise recommendations for clinical diagnosis and treatment. This facilitates discussions between patients and doctors about personalized treatment plans, thereby enhancing shared decision-making (SDM) and improving doctor-patient relationships. However, other scholars express concerns about potential negative effects (14). Dey notes that excessive reliance on medical AI by some patients could lead to detachment from professional doctors, potentially worsening doctor-patient relationships (15). Feng highlights a growing distrust among some patients toward medical AI, which may further damage doctor-patient relationships and increase conflicts (16).

The impact of medical AI chat assistants on treatment outcomes also shows varied perspectives. Kong et al. argue that the machine learning capabilities of medical AI can expand the range of medical resources and accelerate care, which is crucial for addressing issues of insufficient and unevenly distributed medical resources (17). Dey found that integrating ChatGPT into diabetes care enables patients to receive personalized medical guidance and enhances their involvement in managing their condition (15). Conversely, Shaw et al. point out the limitations of AI in healthcare, including potential issues with data quality and privacy, which could lead to incorrect guidance or privacy breaches (18). Triberti et al. discuss the “third-order effect” in psychological terms, where AI involvement in health decisions may lead to decision delays or paralysis, confusion in doctor-patient communication, and ambiguity in responsibility (19).

### 2.2 Application and effects of medical AI chat assistants

#### 2.2.1 Application status and ethical risks

Medical AI chat assistants represent a new form of healthcare technology developed within the context of large language models. These assistants enable patients to provide information such as their age, symptoms, and medical history through conversation, in exchange for receiving a diagnosis and treatment plan. Healthcare professionals can use these AI-generated assessments to validate or assist their own diagnostic and treatment decisions.

In terms of application, Nov et al. conducted a survey to explore respondents’ ability to recognize and trust medical advice provided by ChatGPT compared to human doctors. The findings indicated that ChatGPT’s accuracy in addressing patients’ medical questions ranged from 49 to 85%, with a minimal distinction rate between human and machine feedback (65.5% for humans vs. 65.1% for machines) (9). Moreover, the trust in ChatGPT’s medical responses was moderately positive. Additionally, Ayesha et al. investigated the potential of medical artificial intelligence in assisting healthcare professionals with diagnosing and treating cardiovascular diseases through an experimental approach. They posed 10 hypothetical clinical cardiovascular disease questions to ChatGPT and had the medical feedback evaluated by human medical experts. The results showed that eight of ChatGPT’s responses were accurate diagnoses, while the other two, although not entirely incorrect, had some discrepancies from the actual diagnostic procedures (10).

In terms of ethical risks, Liu highlighted significant concerns regarding the “technological black box” and the autonomous learning capabilities of medical AI. These risks include challenges in regulating the protection of medical data and difficulties in assigning accountability when medical harm occurs (11). On the other hand, Starke et al. developed a trust model based on research into human-machine interaction. They argued that despite the risks, medical AI can be considered trustworthy by focusing on its reliability, capability, and intent. They called for the cultivation of reasonable trust in medical AI (12).

#### 2.2.2 Impact on doctor-patient relationships and treatment outcomes

The impact of medical AI chat assistants on doctor-patient relationships has elicited mixed views within the academic community. Some scholars argue that medical AI chat assistants have a positive influence on doctor-patient relationships. Margam suggests that ChatGPT provides medical professionals with extensive medical knowledge, which helps them make more informed decisions and reduces administrative burdens, enabling them to focus on delivering compassionate and effective patient care (13). Similarly, Lu et al., based on evidence-based medicine principles, argue that AI can offer precise recommendations for clinical diagnosis and treatment. This facilitates discussions between patients and doctors about personalized treatment plans, thereby enhancing shared decision-making (SDM) and improving doctor-patient relationships. However, other scholars express concerns about potential negative effects (14). Dey notes that excessive reliance on medical AI by some patients could lead to detachment from professional doctors, potentially worsening doctor-patient relationships (15). Feng highlights a growing distrust among some patients toward medical AI, which may further damage doctor-patient relationships and increase conflicts (16).

The impact of medical AI chat assistants on treatment outcomes also shows varied perspectives. Kong et al. argue that the machine learning capabilities of medical AI can expand the range of medical resources and accelerate care, which is crucial for addressing issues of insufficient and unevenly distributed medical resources (17). Dey found that integrating ChatGPT into diabetes care allows patients to receive personalized medical guidance and enhances their involvement in managing their condition (15). Conversely, Shaw et al. point out the limitations of AI in healthcare, such as potential issues with data quality and privacy, which could lead to incorrect guidance or privacy breaches (18). Triberti et al. discuss the “third-order effect” in psychological terms, where AI involvement in health decisions might lead to decision delays or paralysis, confusion in doctor-patient communication, and ambiguity in responsibility (19).

### 2.3 Development of the UTAUT model

#### 2.3.1 Application of UTAUT in communication studies

Research on user technology acceptance behavior is extensive, with researchers often building on previous work by incorporating other relevant theories to design various extended new theoretical models for specific application scenarios. The relatively mature technology acceptance models that have been empirically tested include the Technology Acceptance Model 2 (TAM2), the Decomposed Theory of Planned Behavior (DTPB), and the Unified Theory of Acceptance and Use of Technology (UTAUT).

The Technology Acceptance Model 2 (TAM2) extends the original Technology Acceptance Model (TAM). TAM, proposed by Davis, suggests that the attitude toward use (AU) influences behavioral intention to use, which in turn affects actual usage behavior. This attitude is determined by two core variables: perceived usefulness (PU) and perceived ease of use (PEU) (20). Venkatesh and Davis expanded this model to form TAM2 by introducing social influence (SI) and cognitive instrumental processes (CI) as additional variables affecting PU and usage intentions. TAM2 explains 60% of user technology acceptance behavior (21).

The Decomposed Theory of Planned Behavior (DTPB) was developed by Taylor and Todd by expanding upon the Technology Acceptance Model (TAM), Theory of Planned Behavior (TPB), and Theory of Reasoned Action (TRA). The researchers decomposed attitude, subjective norm, and perceived behavioral control from the original models, which clarifies the relationships between factors, making it easier to understand what influences technology usage. DTPB explains 60% of user intention to use, outperforming TAM and TPB (22).

The Unified Theory of Acceptance and Use of Technology (UTAUT) was developed by Venkatesh et al. by integrating key factors from eight user acceptance-related theoretical models, including TAM, TPB, and Innovation Diffusion Theory (IDT). UTAUT posits that four direct factors—Performance Expectancy (PE), Effort Expectancy (EE), Social Influence (SI), and Facilitating Conditions (FC)—determine user acceptance and usage behavior (23). In 2012, Venkatesh et al. refined the model by including Hedonic Motivation, Price Value, and Habit as core variables (24). UTAUT explains 69% of technology adoption intentions and behaviors and is broadly applicable, making it suitable for this study on healthcare professionals’ and patients’ acceptance of medical AI chat assistants.

In the field of health communication, the UTAUT model has been utilized to explain the factors and motivations underlying the adoption of medical products or technologies. For instance, Wang et al. combined UTAUT with the Health Belief Model to establish a model for mask acceptance during the COVID-19 pandemic. Against the backdrop of the COVID-19 pandemic, the researchers employed an extended UTAUT model to validate university students’ adoption behavior and psychological mechanisms toward Learning Management Systems (LMS) across five Asian countries (25).

The study found that social influence, perceived susceptibility, hedonic motivation, and communication barriers significantly impacted the willingness of Chinese people to wear masks. Researchers have noted variations in the impact of UTAUT’s core variables across different scenarios (26). Cao et al. expanded the UTAUT model using Protection Motivation Theory to explain Japanese youth’s acceptance of mobile health (mHealth), finding that Performance Expectancy directly influences behavioral intentions to use mobile health services (27). Conversely, Arfi et al. employed the UTAUT model to investigate factors influencing patients’ use of electronic medical services via the Internet of Things, and found that Performance Expectancy did not significantly affect patients’ intentions to use electronic medical services. Therefore, this study will adapt and extend the UTAUT model based on specific application contexts to identify targeted user intention factors (28).

#### 2.3.2 Integration of artificial intelligence into daily life: acceptance of AI agents

Artificial intelligence agents have emerged across various industries and contexts, and academic research has begun to explore how audiences receive these virtual intelligent agents. Wölker et al. examined the impact of news-writing robots on the journalism industry. Their experimental study investigated readers’ ability to distinguish and trust automated news production, revealing that European readers exhibited equal trust in news articles produced by robots and those produced through traditional methods, with even higher trust in robot-generated sports news. Additionally, trust did not significantly influence readers’ decisions to read or avoid news articles (29).

Building on the Unified Theory of Acceptance and Use of Technology (UTAUT) model, Wang et al. incorporated the “Computers Are Social Actors” (CASA) paradigm, adding variables such as perceived credibility, perceived anthropomorphism, and perceived agency to refine the measurement model (30). They used a questionnaire survey to explore user attitudes and acceptance levels of AI news anchors in the news industry.

Balakrishnan et al. focused on virtual chatbots in the service industry, extending the UTAUT model by incorporating user, system, and social perception factors. Their study found that this extended model provided a better explanation of users’ behavioral intentions under these conditions (31).

In summary, there is currently limited academic attention to the acceptance of medical AI chat assistants as artificial intelligence agents, and research on the acceptance of large language models in the medical field remains scarce.

### 2.4 Conjectural framework and hypotheses development

It is worth noting that the present study undertakes a context-sensitive reduction of the original UTAUT framework. In the high-stakes domain of medical practice—where diagnostic accuracy rather than operational ease is the overriding concern—Effort Expectancy is expected to yield limited explanatory power for adoption intention. Within China’s hospital milieu, policy mandates and departmental protocols exert a far stronger influence on technology uptake than peer pressure; consequently, Social Influence is provisionally omitted. Moreover, Facilitating Conditions are largely institutionally provisioned after initial deployment and are therefore deemed peripheral to first-time use intention, leading to their exclusion from the current model.

#### 2.4.1 Performance expectancy

Building on Venkatesh et al., Performance Expectancy denotes an individual’s perception that using a technology will enhance the attainment of valued outcomes; it is consistently identified as a primary determinant of behavioral intention. In the context of smart healthcare promotion, Alam et al. further demonstrated that patients are more likely to adopt mobile health services when they perceive such technologies as instrumental in improving efficiency (32). Consequently, this study conceptualizes Performance Expectancy as “healthcare professionals’ and patients’ perceived usefulness and anticipated outcomes of medical AI chat assistants,” and posits the following hypothesis:

*H*1: Performance Expectancy (PE) significantly positively influences the Intention to Use medical AI chat assistants (IU).

#### 2.4.2 Perceived cost

Economic barriers are widely recognized as primary obstacles to the diffusion of technologies and services. Park et al. define Perceived Cost as an individual’s tendency to weigh the anticipated benefits of a technology or service against its consumption costs (33). Similarly, Singer et al. demonstrate that patients base their healthcare decisions on the perceived benefits and costs of medical services, underscoring the critical influence of perceived cost on technology adoption (34). Accordingly, this study operationalizes Perceived Cost as “the cost burden associated with using medical AI chat assistants” and formulates the following hypothesis:

*H*2: Perceived Cost (PC) significantly negatively influences the Intention to Use medical AI chat assistants (IU).

#### 2.4.3 Digital access

Fridsma et al. identify digital access as a determinant of a healthy society (35). Yet the digital divide engenders inequities in patients’ digital access and constrains the availability of certain telehealth technologies. Eruchalu et al. note that although the effect of digital-access disparities on COVID-19 mitigation has not been quantitatively examined, unequal digital access in New York City widened gaps in pneumonia-treatment availability (36). These findings underscore the critical influence of digital access on users’ ability to engage with medical technologies and utilize healthcare services. Consequently, this study proposes the following hypothesis:

*H*3: Digital Access (DA) significantly positively influences the Intention to Use medical AI chat assistants (IU).

#### 2.4.4 Digital competence

Zhao et al. conceptualize digital competence as the capacity to acquire and apply knowledge, skills, and cognitive orientations necessary for living and working in the digital realm, which can be progressively developed through sustained social engagement (37). Adopting a market perspective, Malchenko et al. further demonstrate that consumers’ digital competence facilitates their acceptance and utilization of technologies, as well as their participation in value co-creation (38). Accordingly, this study advances the following hypothesis:

*H*4: Digital Competence (DC) has a significantly positive influence on the Intention to Use medical AI chat assistants (IU).

#### 2.4.5 Socioeconomic status and age

Drawing on Ren, socioeconomic status (SES) in the Chinese context is conceived as an aggregate evaluation of an individual’s or household’s economic and social standing, with income, educational attainment, and occupational prestige as its core constituents. SES is calculated here via a simple additive index (see Formula 1). Okunrintemi et al. demonstrate that economic income exerts a direct influence on individuals’ utilization of healthcare technologies: patients with higher incomes enjoy superior medical care and service quality, whereas those with lower incomes report markedly poorer healthcare experiences (39). Age is also a salient determinant; Alameraw et al. employed a modified UTAUT-2 model to demonstrate that younger healthcare professionals exhibit greater openness to remote-monitoring technologies (40, 41). Consequently, this study advances the following hypotheses:

*H*5: Socioeconomic Status (SES) significantly positively influences the Actual Usage Behavior of medical AI chat assistants (AUB).

*H*6: Age (A) significantly negatively influences the Actual Usage Behavior of medical AI chat assistants (AUB).

Formula 1 SES Calculation Method.


(1) SES=Education Score+Income Score+Occupation Score.

#### 2.4.6 Consultation frequency

Ridd et al. operationalize consultation frequency as the number of physician visits within a specified period; this metric gages patients’ demand for healthcare services and serves as an indicator of disease-management effectiveness and treatment outcomes (42). Given heterogeneity in patients’ medical preferences and needs, the influence of consultation frequency on the utilization of alternative consultation modalities remains indeterminate. Given the current low penetration of medical AI chat assistants, higher consultation frequencies may reduce reliance on these tools. Consequently, the impact of consultation frequency on the actual usage behavior of medical AI chat assistants warrants investigation; accordingly, this study advances the following hypothesis:

*H*7: Consultation Frequency (CF) significantly negatively influences the Actual Usage Behavior of medical AI chat assistants (AUB).

#### 2.4.7 Intention to use

Many technology acceptance theories, such as TAM and UTAUT, have validated the relationship between Intention to Use (IU) and Actual Usage Behavior (AUB). Subsequent studies have also confirmed this relationship. For instance, Chen et al. employed an improved UTAUT model to measure factors affecting the use of wearable devices among older adults, hypothesizing that intention to use has a significant positive impact on actual usage behavior (43). Thus, the following hypothesis is proposed:

*H*8: Intention to Use (IU) significantly positively influences the Actual Usage Behavior of medical AI chat assistants (AUB).

**Figure 1:**
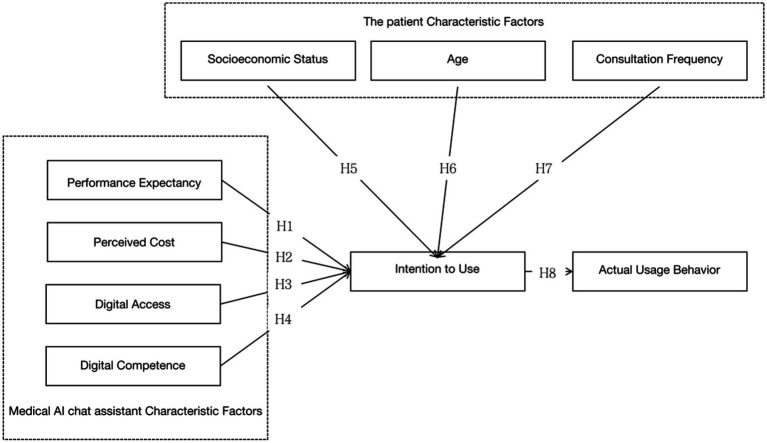
Proposed improved UTAUT in the present study.

In summary, this paper proposes eight hypotheses, where H1, H2, H3, and H4 are related to factors influencing the use of medical AI chat assistants, H5, H6, and H7 pertain to patient characteristics, and H8 concerns the impact of intention to use on the actual usage behavior of medical AI chat assistants (Figure 1).

Hypotheses Related to Factors Influencing the Use of Medical AI Chat Assistants:

*H*1: Performance Expectancy (PE) positively influences the intention to use (IU) medical AI chat assistants.

*H*2: Perceived Cost (PC) negatively influences the intention to use (IU) medical AI chat assistants.

*H*3: Digital Access (DA) positively influences the intention to use (IU) medical AI chat assistants.

*H*4: Digital Competence (DC) positively influences the intention to use (IU) medical AI chat assistants.

Hypotheses Related to Patient Characteristics:

*H*5: Patients’ Socioeconomic Status (SES) positively influences the actual usage behavior (AUB) of medical AI chat assistants.

*H*6: Patients’ Age (A) negatively influences the actual usage behavior (AUB) of medical AI chat assistants.

*H*7: Consultation Frequency (CF) negatively influences the actual usage behavior (AUB) of medical AI chat assistants.

*H*8: Intention to Use (IU) positively influences the actual usage behavior (AUB) of medical AI chat assistants.

## 3 Research methodology

### 3.1 Research design

Anchored in the extended Unified Theory of Acceptance and Use of Technology (UTAUT), this study employs a cross-sectional survey design to capture the differential acceptance logics of medical AI chatbots among Chinese physicians and patients within a single temporal slice.

### 3.2 Measurement instruments

The questionnaire is bifurcated into two segments. Section A elicits demographic attributes—region, age, and outpatient-visit frequency—while Section B operationalizes six reflective constructs: performance expectancy (PE; 4 items), perceived cost (PC; 3 items), digital access (DA; 4 items), digital competence (DC; 4 items), intention to use (IU; 3 items), and actual use behavior (UB; 2 items). All items are measured on a five-point Likert continuum (1 = strongly disagree, 5 = strongly agree). Adapted from canonical scales advanced by Venkatesh et al. (23), Dehghani et al. (44), Guo (45), Chen (46), and Mariani et al. (47), the measures underwent bilingual back-translation and cultural decentering to ensure semantic equivalence in the Chinese healthcare context. The complete item battery is presented in Appendix A. A pilot test (*n* = 34) yielded Cronbach’s *α* = 0.943 and KMO = 0.743 (*p* < 0.001), attesting to internal consistency and sampling adequacy.

### 3.3 Sampling frame

The target population consists of mainland Chinese residents aged 15 or older who have engaged with a medical AI chatbot at least once within the preceding 12 months.

### 3.4 Sampling strategy

Quota sampling was deployed along six demographic vectors calibrated to national benchmarks: gender (male 51%, female 49%), residence (urban 66%, rural 34%), age (≤15 1.8%, 16–59 61%, ≥60 21%), education (primary or below 5%, junior high 7%, senior high 3%, university or above 3%), income (five equal quintiles), and region (eastern 40%, central 26%, western 27%, northeastern 7%). The first five quotas align with the 2023 National Economic Performance release (48); the regional quotas are derived from the Third Communique of the Seventh National Population Census (49).

### 3.5 Data collection procedure

Fieldwork was commissioned to the professional agency “Survey Factory” and executed from March 10 to April 28, 2024, via a hybrid online-offline protocol. Invalid cases were identified and expunged through attention-check items and response latency thresholds, resulting in 500 valid responses. The achieved sample exhibits proportional congruence with the predefined demographic quotas; 77% of respondents reported 1–5 outpatient visits in the past year, while 2% reported more than 10 visits.

### 3.6 Estimation techniques

Reliability and validity diagnostics, together with correlational and difference tests, will be performed in SPSS 29.0. A covariance-based structural equation model (SEM) will be estimated in AMOS 28.0 to assess the nomological validity of the extended UTAUT framework across the physician-patient dyad.

## 4 Results

### 4.1 Reliability and validity testing

Reliability reflects the consistency of measurement results. The reliability was assessed using Cronbach’s alpha, which yielded a value of 0.927 (>0.8), indicating that the research results are reliable. Validity reflects the accuracy of the measurement results. To test the structural validity of the questionnaire scales, exploratory factor analysis was conducted. The Kaiser-Meyer-Olkin (KMO) measure of sampling adequacy was 0.939 (>0.5), and Bartlett’s test of sphericity showed a *p*-value <0.001 (<0.05), indicating good structural validity.

### 4.2 Correlation analysis

Table 1 summarizes the correlations between Performance Expectancy, Perceived Cost, Digital Access, Digital Competence, and Intention to Use, as well as the correlation between Socioeconomic Status, Intention to Use, and Actual Usage Behavior. Pearson correlation analysis was used to measure the strength of these correlations. The analysis shows that Performance Expectancy, Perceived Cost, Digital Access, and Digital Competence are all significantly correlated with Intention to Use, with *p*-values < 0.001 (*p* < 0.05). The correlation coefficients are 0.532, 0.540, 0.495, and 0.497, respectively, indicating positive correlations between these factors and Intention to Use. Additionally, Intention to Use is a significant factor influencing Actual Usage Behavior, with a *p*-value < 0.001 (*p* < 0.05) and a correlation coefficient of 0.522, which is greater than 0, indicating a positive correlation between Intention to Use and Actual Usage Behavior. Socioeconomic Status (*p* = 0.42 > 0.05) does not have a significant correlation with Actual Usage Behavior.

**Table 1:** Correlations analysis.

Pearson		Performance expectancy	Perceived cost	Digital access	Digital competence	Intention to use	Socioeconomic status	Actual usage behavior
Performance expectancy	Pearson Correlation	1						
	Significance (two-tailed)							
Perceived cost	Pearson Correlation	0.439^**^	1					
	Significance (two-tailed)	<0.001						
Digital access	Pearson Correlation	0.431^**^	0.507^**^	1				
	Significance (two-tailed)	<0.001	<0.001					
Digital competence	Pearson Correlation	0.429^**^	0.441^**^	0.499^**^	1			
	Significance (two-tailed)	<0.001	<0.001	<0.001				
Intention to use	Pearson Correlation	0.532^**^	0.540^**^	0.495^**^	0.497^**^	1		
	Significance (two-tailed)	<0.001	<0.001	<0.001	<0.001			
Socioeconomic status	Pearson Correlation	0.011	−0.019	0.046	0.05	0.041	1	
	Significance (two-tailed)	0.799	0.673	0.299	0.266	0.357		
Actual usage behavior	Pearson Correlation	0.602^**^	0.429^**^	0.455^**^	0.500^**^	0.522^**^	0.036	1
	Significance (two-tailed)	<0.001	<0.001	<0.001	<0.001	<0.001	0.42	

**the correlation is significant at the 0.01 level (two-tailed).

Table 2 summarizes the correlations between Patient Age, Consultation Frequency, Income Level, Education Level, and Occupation Status with Actual Usage Behavior, using Spearman’s rank correlation analysis to measure the strength of these correlations. The analysis shows that Patient Age and Consultation Frequency are significant factors affecting Actual Usage Behavior, with *p*-values < 0.05. The correlation coefficients are 0.120 and 0.143, respectively, indicating positive correlations between these factors and Actual Usage Behavior. Income Level, Education Level, and Occupation Status (*p* = 0.097 > 0.05; *p* = 0.067 > 0.05; *p* = 0.284 > 0.05) do not have significant correlations with Actual Usage Behavior.

**Table 2:** Correlations analysis.

Spearman’s Rho		Age	Consultation frequency	Income	Education	Occupation	Actual usage behavior
Age	Correlation Coefficient	1					
	Significance (two-tailed)	.					
Consultation frequency	Correlation Coefficient	0.134^**^	1				
	Significance (two-tailed)	0.003	.				
Income	Correlation Coefficient	0.377^**^	0.028	1			
	Significance (two-tailed)	<0.001	0.534	.			
Education	Correlation Coefficient	−0.138^**^	−0.144^**^	0.370^**^	1		
	Significance (two-tailed)	0.002	0.001	<0.001	.		
Occupation	Correlation Coefficient	−0.214^**^	−0.066	0.345^**^	0.601^**^	1	
	Significance (two-tailed)	<0.001	0.139	<0.001	<0.001	.	
Actual usage behavior	Correlation Coefficient	0.120^**^	0.143^**^	0.074	−0.082	0.048	1
	Significance (two-tailed)	0.007	0.001	0.097	0.067	0.284	.

**the correlation is significant at the 0.01 level (two-tailed).

### 4.3 Confirmatory factor analysis

Confirmatory factor analysis was conducted through structural equation modeling to assess the aggregation of items within variables. Post-hoc power analysis (G*Power 3.1.9.7) indicated that, assuming *α* = 0.05, f^2^ = 0.10, 1-*β* = 0.90, and six predictors, a minimum of 119 participants was required. Our sample of 500 exceeds this threshold, safeguarding the stability of parameter estimates and the interpretive fidelity of the structural model. As shown in Table 3, the model fit indices including CMIN/DF (1.558 < 3), RMSEA (0.033 < 0.08), GFI (0.950 > 0.9), NFI (0.950 > 0.9), TLI (0.977 > 0.9), and CFI (0.981 > 0.9) all met the evaluation criteria for model fitting, indicating a good fit of the model. In this model, the *p*-value of the significant variables (0.000) is less than 0.05, indicating significant correlations between manifest variables and latent variables, and suggesting that these manifest variables can explain their corresponding latent variables. As shown in Table 4, the Average Variance Extracted (AVE) values of latent variables are greater than 0.5, and the composite reliability values are greater than 0.8, indicating that the scale has good composite reliability.

**Table 3:** Model fit test.

Model	*p*	CMIN/DF	RMSEA	GFI	NFI	TLI	CFI
					Delta 1	Rho 2	
Index of fit	0.000	1.558	0.033	0.950	0.950	0.977	0.981

**Table 4:** Convergence validity and composite reliability of the scale dimensions.

Path relationship	Estimate	AVE	CR
PE1←PE	0.780	0.635	0.839
PE2←PE	0.768		
PE3←PE	0.841		
PC1←PC	0.755	0.609	0.824
PC2←PC	0.774		
PC3←PC	0.812		
DA1←DA	0.788	0.586	0.809
DA2←DA	0.721		
DA3←DA	0.785		
DC1←DC	0.762	0.568	0.840
DC2←DC	0.814		
DC3←DC	0.655		
DC4←DC	0.775		
IU1←IU	0.760	0.517	0.842
IU2←IU	0.717		
IU3←IU	0.692		
IU4←IU	0.722		
IU5←IU	0.702		
AUB1←AUB	0.802	0.678	0.863
AUB2←AUB	0.829		
AUB3←AUB	0.839		

**Figure 2:**
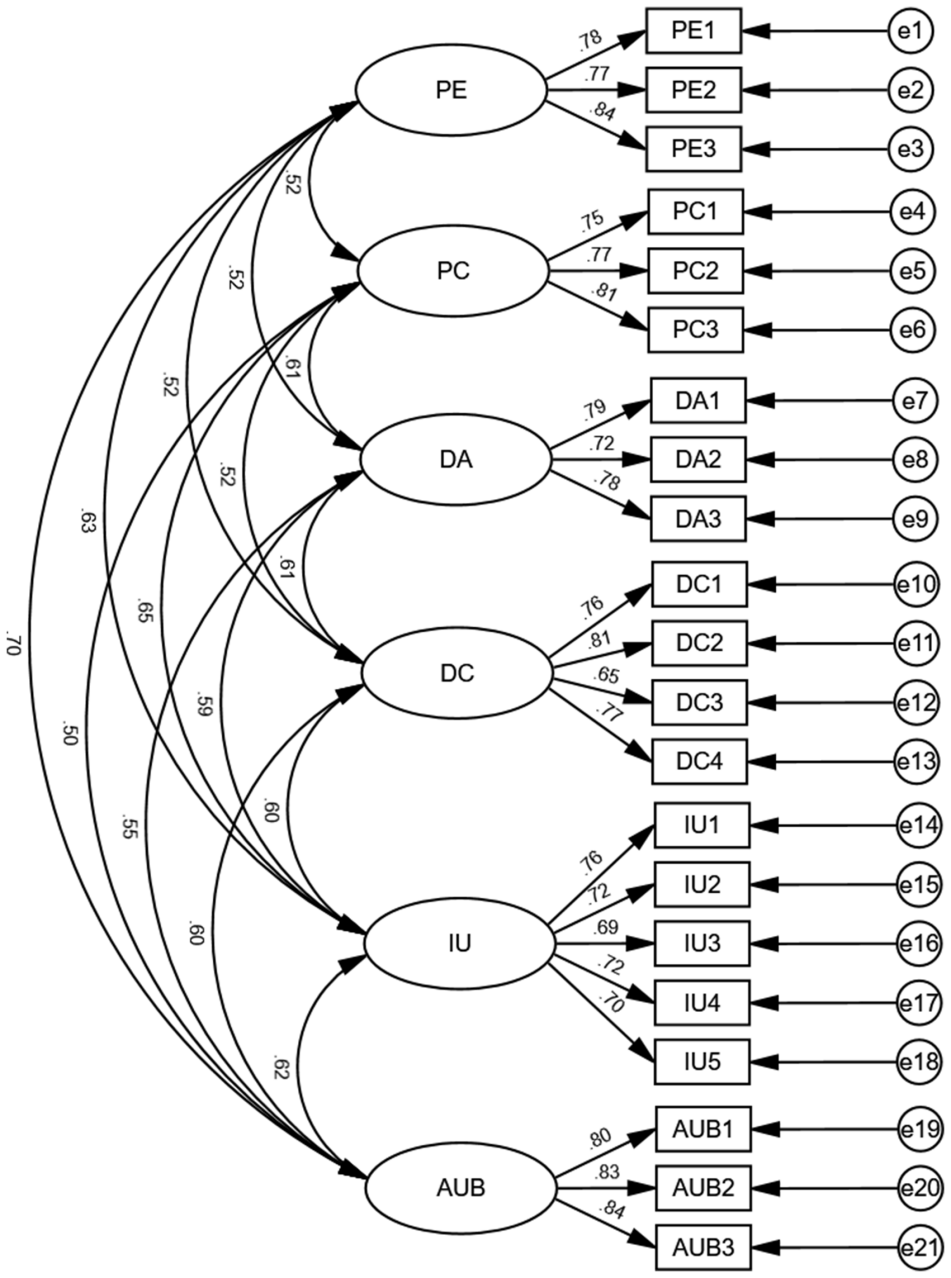
Confirmatory factor analysis model diagram.

Furthermore, the discrimination between variables is effective when the correlation coefficients between variables are less than the square root of their average variance. According to Table 5, the correlation coefficients of the main variables are less than their respective average variance square roots. Therefore, it can be determined that there is good discriminant validity between different latent variables. The specific confirmatory factor analysis model for variable items is shown in Figure 2.

**Table 5:** Discrimination validity of the scales.

Variable	PE	PC	DA	DC	IU	AUB
PE	0.635					
PC	0.523	0.609				
DA	0.525	0.610	0.586			
DC	0.517	0.522	0.608	0.568		
IU	0.632	0.650	0.592	0.601	0.517	
AUB	0.701	0.505	0.548	0.603	0.621	0.678
√AVE	0.797	0.781	0.765	0.754	0.719	0.823

### 4.4 Hypothesis testing

#### 4.4.1 Model fitting

This study aims to discuss the mechanisms of influence factors on the usage intentions and behaviors of medical AI chat assistants. Therefore, a structural equation model was constructed using Amos software to examine the path relationships among Performance Expectancy, Perceived Cost, Digital Access, and Digital Competence. According to Table 6, the model fit indices—CMIN/DF (2.074 < 3), RMSEA (0.046 < 0.08), GFI (0.933 > 0.9), NFI (0.932 > 0.9), TLI (0.956 > 0.9), CFI (0.963 > 0.9)—all met the criteria, with the *p*-value of significant variables (0.000) being less than 0.05. Therefore, the model demonstrates good absolute fit and is reliable.

**Table 6:** The fit of the structural equation model.

Model	*p*	CMIN/DF	RMSEA	GFI	NFI	TLI	CFI
					Delta 1	Rho 2	
Default model	0.000	2.074	0.046	0.933	0.932	0.956	0.963

#### 4.4.2 Testing the effects of path relationships

**Figure 3:**
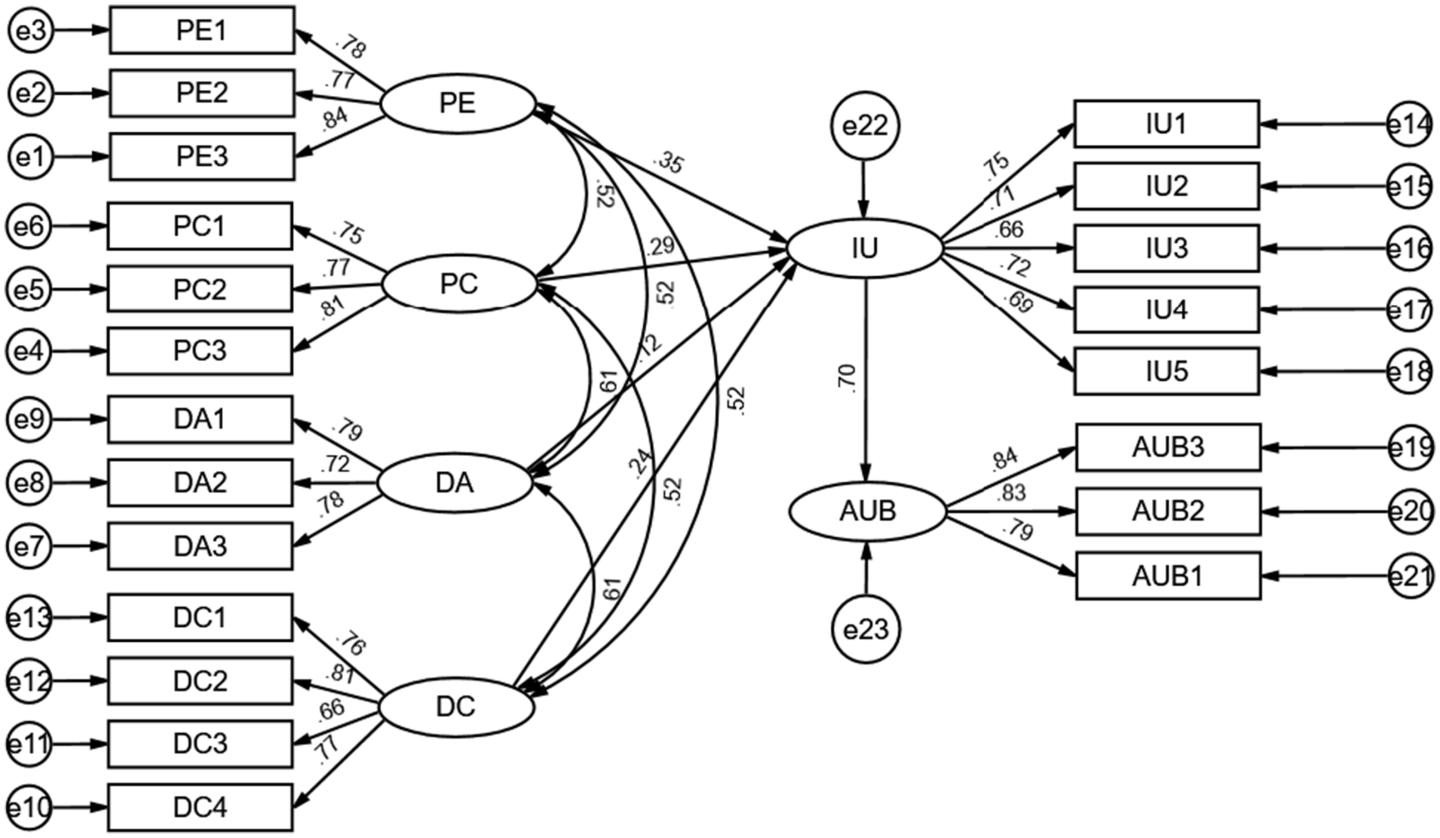
Structural equation model.

The results of testing the effects of path relationships with Amos are displayed in Table 7, and the structural equation model is illustrated in Figure 3. Performance Expectancy, Perceived Cost, Digital Access, and Digital Competence have significant positive effects on the intention to use medical AI chat assistants (*β* = 0.354, *p* < 0.05; *β* = 0.286, *p* < 0.05; *β* = 0.120, *p* < 0.05; *β* = 0.239, *p* < 0.05). These results confirm Hypotheses H1, H3, and H4, while Hypothesis H2 is not supported; the actual direction of the impact of Perceived Cost on the intention to use medical AI chat assistants contradicts the hypothesis. The intention to use has a significant positive impact on the usage behavior of medical AI chat assistants (*β* = 0.699, *p* < 0.05), thereby validating Hypothesis H8.

**Table 7:** Analysis and test of acting path.

Acting path	Estimate	S.E.	C.R.	*p*
IU←PC	0.286	0.05	5.036	***
IU←DC	0.239	0.05	4.359	***
IU←PE	0.354	0.045	6.704	***
IU←DA	0.120	0.051	1.990	0.047
AUB←IU	0.699	0.072	13.142	***

### 4.5 Analysis of variance

A one-way ANOVA was conducted to analyze the differences in usage behavior of medical AI chat assistants among patients of different ages. The test for homogeneity of variances was passed (see Appendix 2). The results of the ANOVA are presented in Table 8, indicating significant differences in usage behavior across different age groups (*F* = 3.296, *p* < 0.05). This suggests that there are significant age-related differences in usage behavior. Further multiple comparisons (see Appendix 2) revealed that patients aged 16–30 are the least likely to engage in usage behavior compared to other age groups.

**Table 8:** Analysis of variance.

Source	ANOVA for usage behavior				
	Sum of squares	Degrees of freedom	Mean square	F	Significance
Between Groups	10.839	4	2.71	3.296	0.011
Within Groups	406.957	495	0.822		
Total	417.796	499			

A one-way ANOVA was conducted to analyze differences in the usage behavior of medical AI chat assistants based on the consultation frequency. The test for homogeneity of variances was not passed (see Appendix 3), so a non-parametric test was employed for the analysis. The analysis was conducted using the Kruskal-Wallis test, and the results are shown in Table 9, indicating a Kruskal-Wallis H statistic of 12.157, with *p* = 0.007 < 0.05. This indicates significant differences in usage behavior among patients with different consultation frequencies. Further pairwise comparisons (see Appendix 3) revealed differences in the usage behavior of medical AI chat assistants among groups with 0–10 visits. It was found that as the consultation frequency increases, patients are more likely to engage in usage behavior.

**Table 9:** Kruskal-Wallis H test.

Independent samples Kruskal-Wallis test^a,b^	
Kruskal-Walli H	12.157
Degrees of freedom	3
Asymptotic significance	0.007

^a^Kruskal-Wallis Test; ^b^Grouping Variable: Consultation Frequency.

## 5 Discussion

This study finds that performance expectancy, perceived cost, digital access, and digital competence positively influence the intention to use medical AI chat assistants, whereas patient age, consultation frequency, and intention positively affect actual usage behavior. In addition, socioeconomic status is not correlated with usage behavior. The hypothesis testing results are shown in Table 10.

**Table 10:** Results of hypothesis testing.

Hypothesis	Result	Additional information
H1: Performance Expectancy positively influences the intention to use medical AI chat assistants.	Supported	
H2: Perceived Cost negatively influences the intention to use medical AI chat assistant.	Not Supported	Positive influence
H3: Digital Access positively influences the intention to use medical AI chat assistants.	Supported	
H4: Digital Competence positively influences the intention to use medical AI chat assistants.	Supported	
H5: Patients’ socioeconomic status positively influences the usage behavior of medical AI chat assistants.	Not Supported	Not related
H6: Patients’ age negatively influences the actual usage behavior of medical AI chat assistants.	Not Supported	Positive influence
H7: Consultation frequency negatively influences the actual usage behavior of medical AI chat assistants.	Not Supported	Positive influence
H8: Intention to use positively influences the actual usage behavior of medical AI chat assistants.	Supported	

First, this study confirms that performance expectancy, digital access, and digital competence are positively associated with the intention of physicians and patients to use medical AI chat assistants. Moreover, the intention to use these assistants has a positive impact on actual usage behavior. Among these, the positive effect of performance expectancy on intention is consistent with previous findings (23), indicating that the perceived usefulness and functional value of medical AI chat assistants are key determinants of willingness to use.

Second, Park et al. suggest that patients make medical decisions based on perceived benefits and costs, with lower expected costs leading to a stronger intention (33). However, physicians and patients in this study exhibit the opposite attitude toward the expected cost burden of using medical AI chat assistants, showing that as expected costs increase, intention to adopt also increases. One reason is that Chinese physicians and patients generally hold a high level of agreement that more expensive medical products are more effective. Wang et al. found that 82.08% of patients believe that higher-priced medical products are superior to lower-priced ones, associating price level with product quality and interpreting high prices as symbols of advanced technology and quality assurance (50). In addition, current mainstream medical AI chatbots in China commonly adopt a “basic free + premium paid” pricing strategy, making it difficult for users to perceive the true marginal cost. Due to information asymmetry, users equate “high price” with “high value” and no longer view perceived cost as an economic burden but as an external signal of technological credibility. These conditions result in a positive correlation between willingness to use and perceived cost.

Third, this study points out the development potential of medical AI chat assistants in an aging society. Results confirm a positive correlation between patient age and consultation frequency, as well as intention to use medical AI chat assistants. Li et al. found that as patient age increases and offline consultation frequency rises, the intention to use medical technology products decreases (51). However, this study finds that medical AI chat assistants are more attractive to older patients and those who frequently visit offline. These findings align with those of Zhang et al., who showed that as age increases, Chinese patients place greater emphasis on convenience and service attitude in healthcare (52).

Finally, this study refutes the common assumption in technology diffusion literature that patients’ socioeconomic status is positively correlated with healthcare behavior. Zhang et al. found that generally income and education levels positively influence healthcare behavior (52). However, this study finds no significant impact of socioeconomic status on the intention to use medical AI chat assistants, and factors such as income and educational level are not correlated with this intention.

## 6 Implications

Theoretically, this study extends the applicability of the Unified Theory of Acceptance and Use of Technology (UTAUT) within digital-health contexts. By integrating perceived cost, digital access, digital competence, and consultation frequency into the original framework, we provide an expanded model that clarifies the mechanisms underlying intention and behavior toward medical AI chat assistants.

Practically, as these tools rely on web or mobile APIs and H5 interfaces, designers should prioritize usability and cognitive load reduction to deliver low-threshold, age-friendly products (53). Pricing policies should align with China’s medical-service pricing mechanisms, avoid excessive charges, and undergo regular cost accounting to ensure market-appropriate fees (54).

Policy-wise, governments should narrow the digital divide by offering data-fee exemptions for health apps, embedding “AI health literacy” modules within national essential public-health services, deploying “silver-age digital coaches,” and including compliant AI consultations in the medical reimbursement catalog to lower economic barriers and incentivize sustained use.

## 7 Conclusion

The integration of artificial intelligence into healthcare is reshaping physicians’ workflows and patients’ care experiences. This study confirms that performance expectancy, perceived cost, digital access, and digital competence serve as critical antecedents to the adoption of medical AI chat assistants by both doctors and patients, while age, consultation frequency, and intention directly drive actual usage. The extended UTAUT framework effectively explains these mechanisms and offers an actionable theoretical and empirical pathway for the equitable diffusion of AI medical technologies in aging societies.

Notably, the findings reveal the unique downward compatibility of medical AI chat assistants: they provide high-quality consultation and diagnostic services at low cost, proving particularly effective for minor ailments and chronic disease management. For developing countries like China, the widespread deployment of these assistants can help mitigate geographical and wealth-based disparities in healthcare resources, reduce the caseload pressure on offline medical institutions, and rebalance the supply and demand within the healthcare system. Moreover, as population aging accelerates, AI assistants offer older adults a more convenient and accessible channel for seeking care, helping them navigate age-related functional limitations and ensuring timely medical support.

## 8 Limitations and potential areas of future studies

Despite revealing significant findings, this study has limitations. First, the influence of the Chinese and Western medical systems should be considered in the scope of factors affecting medical AI chat assistants; however, this is limited by the lack of comprehensive digital resources for traditional Chinese medicine, which leaves current large medical models predominantly Western. Second, the empathic capabilities of medical AI chat assistants should be measured. Empathic communication in doctor-patient interactions can encourage patients to discuss their conditions more openly, improving both the relationship and the quality of medical care (55). In interactions between medical AI chat assistants and patients, patients’ emotions and behaviors can be dynamically perceived and immediately responded to, thus potentially enhancing patients’ willingness to use through empathic communication (56). This aspect was not covered in the current study and needs further development. Looking forward, we invite extensions that enrich the utilitarian core of UTAUT with relational constructs salient to human–AI interaction in clinical settings. Specifically, agent trust and social presence—anchored in the Computers Are Social Actors (CASA) paradigm—promise to illuminate the socio-emotional circuitry that complements instrumental drivers of technology acceptance. The development of large medical AI models is a global endeavor, and implementing this technology in practice requires consideration of different national healthcare systems and policies. Future research should continue to center on developing countries, integrating qualitative data and real-world usage indicators—such as patient satisfaction, diagnostic accuracy, and service trust—to examine how large-scale AI models can advance patient experience within diverse healthcare systems.

## Data Availability

The datasets presented in this study can be found in online repositories. The dataset is available in the Harvard Dataverse repository under DOI 10.7910/DVN/SLWWAG.
