# Supplementary material for: Factors influencing the acceptance of medical AI chat assistants among healthcare professionals and patients: a survey-based study in China
Source: Front Public Health. 2025 Sep 4;13:1637270. doi: 10.3389/fpubh.2025.1637270 (PMC12443681; doi:10.3389/fpubh.2025.1637270)
Supplement: Supplementary file 1 [file Supplementary_file_1.docx]

Supplementary Material

**Appendix**

**Appendix 1. Variable Reference Scale**

| Performance Expectancy  (PE) | PE1: I think the medical AI chat assistant is useful for me. |
| --- | --- |
|  | PE2: I believe using the medical AI chat assistant is more efficient. |
|  | PE3: I think using the medical AI chat assistant helps me solve health issues faster. |
| Perceived Cost  (PC) | PC1: There are financial barriers to using the medical AI chat assistant. |
|  | PC2: I think the cost of using the medical AI chat assistant is high. |
|  | PC3: Overall, using the medical AI chat assistant costs me a lot of money. |
| Digital Access  (DA) | DA1: I have the appropriate electronic devices to use the medical AI chat assistant. |
|  | DA2: I have a reliable internet connection to use the medical AI chat assistant. |
|  | DA3: I am able to find a platform to use the medical AI chat assistant. |
| Digital Competence  (DC) | DC1: I find it easy to locate the platform to use the medical AI chat assistant. |
|  | DC2: When I want to consult the medical AI chat assistant about health issues, I know how to proceed. |
|  | DC3: I verify the answers provided by the medical AI chat assistant before trusting them. |
|  | DC4: I can use the medical AI chat assistant to promote my health. |
| Intention to Use (IU) | IU1: I want to use the medical AI chat assistant. |
|  | IU2: I will choose to use the medical AI chat assistant when needed. |
|  | IU3: If the medical AI chat assistant meets my expectations, I am willing to use it frequently. |
|  | IU4: I think using the medical AI chat assistant for online consultations is more convenient and effective. |
|  | IU5: Relevant organizations should vigorously develop and promote medical AI chat assistants. |
| Actual Usage Behavior (AUB) | AUB1: I already frequently use the medical AI chat assistant. |
|  | AUB2: I would recommend others to use the medical AI chat assistant. |

**Appendix 2. Test for Homogeneity of Variances&Multiple Comparisons**

| Test for Homogeneity of Variances | | | | | |
| --- | --- | --- | --- | --- | --- |
| Test for Homogeneity of Variances | | Levene's Statistic | df1 | df2 | Significance |
| Usage Behavior | Based on Mean | 1.439 | 4 | 495 | 0.22 |

Multiple Comparisons Dependent Variable: Usage Behavior

| Multiple Comparisons | (I) Age | (J) Age | Mean Difference (I-J) | Standard Error | Significance | 95% Confidence Interval | |
| --- | --- | --- | --- | --- | --- | --- | --- |
|  |  |  |  |  |  | Lower Limit | Upper  Limit |
| LSD | Under 15 | 16-30 years | 0.2505 | 0.1311 | 0.057 | -0.007 | 0.508 |
|  |  | 31-45 years | -0.0403 | 0.1311 | 0.759 | -0.298 | 0.217 |
|  |  | 46-59 years | -0.1481 | 0.1305 | 0.257 | -0.405 | 0.108 |
|  |  | 60+ years | -0.1449 | 0.1311 | 0.270 | -0.403 | 0.113 |
|  | 16-30 years | Under 15 | -0.2505 | 0.1311 | 0.057 | -0.508 | 0.007 |
|  |  | 31-45 years | -.2908^*^ | 0.127 | 0.022 | -0.540 | -0.041 |
|  |  | 46-59 years | -.3987^*^ | 0.1264 | 0.002 | -0.647 | -0.15 |
|  |  | 60+ years | -.3954^*^ | 0.127 | 0.002 | -0.645 | -0.146 |
|  | 31-45 years | Under 15 | 0.0403 | 0.1311 | 0.759 | -0.217 | 0.298 |
|  |  | 16-30 years | .2908^*^ | 0.127 | 0.022 | 0.041 | 0.54 |
|  |  | 46-59 years | -0.1078 | 0.1264 | 0.394 | -0.356 | 0.14 |
|  |  | 60+ years | -0.1046 | 0.127 | 0.411 | -0.354 | 0.145 |
|  | 46-59 years | Under 15 | 0.1481 | 0.1305 | 0.257 | -0.108 | 0.405 |
|  |  | 16-30 years | .3987^*^ | 0.1264 | 0.002 | 0.15 | 0.647 |
|  |  | 31-45 years | 0.1078 | 0.1264 | 0.394 | -0.14 | 0.356 |
|  |  | 60+ years | 0.0033 | 0.1264 | 0.979 | -0.245 | 0.252 |
|  | 60+ years | Under 15 | 0.1449 | 0.1311 | 0.27 | -0.113 | 0.403 |
|  |  | 16-30 years | .3954^*^ | 0.127 | 0.002 | 0.146 | 0.645 |
|  |  | 31-45 years | 0.1046 | 0.127 | 0.411 | -0.145 | 0.354 |
|  |  | 46-59 years | -0.0033 | 0.1264 | 0.979 | -0.252 | 0.245 |
| *. The significance level for mean differences is 0.05. | | | | | | | |

**Appendix 3. Test for Homogeneity of Variances&Pairwise Comparisons**

Test for Homogeneity of Variances

| Test for Homogeneity of Variances | | Levene's Statistic | df1 | df2 | Significance |
| --- | --- | --- | --- | --- | --- |
| Usage Behavior | Based on Mean | 4.413 | 3 | 496 | 0.04 |

Pairwise Comparisons for Consultation Frequency

| Sample 1-Sample 2 | Test Statistic | Standard Error | Standard Test Statistic | Significance | Adjusted Significance ^a^ |
| --- | --- | --- | --- | --- | --- |
| 1-4 | -60.386 | 51.147 | -1.181 | 0.238 | 1 |
| 1-2 | -61.302 | 24.937 | -2.458 | 0.014 | 0.084 |
| 1-3 | -102.001 | 29.418 | -3.467 | <.001 | 0.003 |
| 4-2 | 0.916 | 45.831 | 0.020 | 0.984 | 1 |
| 4-3 | 41.614 | 48.415 | 0.860 | 0.390 | 1 |
| 2-3 | -40.699 | 18.705 | -2.176 | 0.030 | 0.177 |
| Each row tests the null hypothesis that the distribution of "Sample 1 and Sample 2" is the same. | | | | | |
| The significance is shown for two-sided tests with a significance level set at .050. | | | | | |
| a. Significance values have been adjusted for multiple tests using the Bonferroni correction method. | | | | | |

**
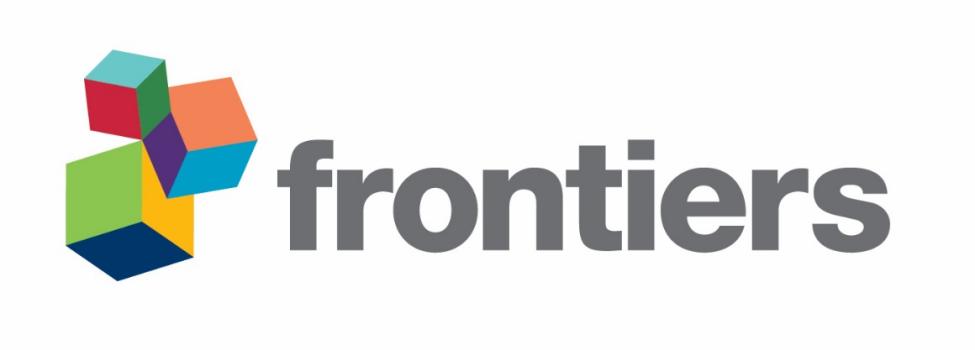
**
